# Direct PCR of indigenous and invasive mosquito species: a time‐ and cost‐effective technique of mosquito barcoding

**DOI:** 10.1111/mve.12154

**Published:** 2015-12-12

**Authors:** A. WERBLOW, E. FLECHL, S. KLIMPEL, C. ZITTRA, K. LEBL, K. KIESER, A. LACINY, K. SILBERMAYR, C. MELAUN, H.‐P. FUEHRER

**Affiliations:** ^1^ Institute for Ecology, Evolution and Diversity, Goethe University (GU); Senckenberg Biodiversity and Climate Research Centre (BiK‐F); Senckenberg Gesellschaft für Naturforschung (SGN)Frankfurt am MainGermany; ^2^ Institute of Parasitology, Department of PathobiologyUniversity of Veterinary Medicine ViennaViennaAustria; ^3^ Institute for Veterinary Public Health, Department for Farm Animals and Veterinary Public HealthUniversity of Veterinary Medicine ViennaViennaAustria

**Keywords:** Barcoding, direct PCR, molecular differentiation, molecular identification, mosquitoes, vector‐borne diseases

## Abstract

Millions of people die each year as a result of pathogens transmitted by mosquitoes. However, the morphological identification of mosquito species can be difficult even for experts. The identification of morphologically indistinguishable species, such as members of the Anopheles maculipennis complex (Diptera: Culicidae), and possible hybrids, such as Culex pipiens pipiens/Culex pipiens molestus (Diptera: Culicidae), presents a major problem. In addition, the detection and discrimination of newly introduced species can be challenging, particularly to researchers without previous experience. Because of their medical importance, the clear identification of all relevant mosquito species is essential. Using the direct polymerase chain reaction (PCR) method described here, DNA amplification without prior DNA extraction is possible and thus species identification after sequencing can be achieved. Different amounts of tissue (leg, head; larvae or adult) as well as different storage conditions (dry, ethanol, −20 and −80 °C) and storage times were successfully applied and showed positive results after amplification and gel electrophoresis. Overall, 28 different indigenous and non‐indigenous mosquito species were analysed using a gene fragment of the COX1 gene for species differentiation and identification by sequencing this 658‐bp fragment. Compared with standard PCR, this method is time‐ and cost‐effective and could thus improve existing surveillance and control programmes.

## Introduction

**Figure 1:**
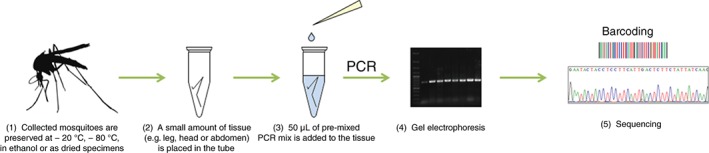
Test procedure for the identification of mosquitoes using direct polymerase chain reaction (PCR).

Every year, around a billion people worldwide become infected with vector‐borne diseases such as malaria, dengue fever, yellow fever and West Nile virus (WNV) (Hill *et al*., 2005; WHO, 2014), and more than 1.3 million people die as a result of such diseases, of which mosquitoes represent the most important vectors (Beerntsen *et al*., 2000). Globally, more than 3500 mosquito species are described. Of these, 50 or 51 species occur in Germany and 43 species have been recorded in Austria (Zittra & Waringer, 2013; Becker *et al*., 2014). In addition to *Culiseta longiareolata* (Diptera: Culicidae), another species has now been documented as newly introduced to Austria (Zittra *et al*., 2014). Climate change and a higher degree of globalization lead to the introduction and spread of species to new regions, countries and even continents (Keller *et al*., 2011). In recent years, the spread of invasive non‐indigenous species, such as *Hulecoeteomyia japonica japonica* (syn. *Ochlerotatus japonicus japonicus*) (Theobald 1901) (Diptera: Culicidae) and *Stegomyia albopicta* (= *Aedes albopictus*) (Skuse 1895) (Diptera: Culicidae), as well as *Stegomyia aegypti* (= *Aedes aegypti*) (Linnaeus 1762), to European countries has increased dramatically (Almeida *et al*., 2007; ECDPC, 2014). All of these species are known to be vectors of a variety of disease agents in the wild (CDCP, 2012; Schaffner *et al*., 2012) and have been proven to possess vector abilities under laboratory conditions. In particular, *St. albopicta* is known to be a vector of 22 arboviruses (Petrić *et al*., 2014). In addition to non‐indigenous species, native species in Austria and Germany can also act as competent vectors for diseases including Sindbis virus, Ockelbo virus, Usutu virus, Batai virus or malaria and WNV (Jöst *et al*., 2010, 2011a, 2011b; Ventim *et al*., 2012; Avšič‐Županc, 2013). Species such as *Culex pipiens*, Linnaeus 1758, *Culex modestus*, Ficalbi 1889 and *Coquillettidia richiardii* (Ficalbi 1889) (Diptera: Culicidae) are considered as the major vectors of WNV in European outbreaks (Votýpka *et al*., 2008). Because of their major impact on health, as well as their ecological and socioeconomic relevance, the clear identification of the different species is essential. However, this is not always possible as morphological identification keys often refer to only certain developmental stages or characteristics that are easily lost during collection or transportation, such as scales and setae. Additionally, morphological identification can be challenging as a result of sibling species complexes, which are often morphologically indistinguishable but are vectors for different pathogens (Kumar *et al*., 2007; Becker *et al*., 2014). For instance, within the *Anopheles maculipennis s.l*. complex, which consists of more than a dozen different species worldwide and eight in Europe, various members are indicated as vectors for different *Plasmodium* species causing malaria in humans. The only distinguishable characteristic is the pigmentation of the egg chorions of the different species. Members of the *Cx. pipiens* complex are distinguishable by the structure of the male genitalia, whereas females of *Cx. pipiens* and *Culex torrentium*, Martini 1925 are difficult to distinguish by morphometric wing characters and identification by the characteristics named requires a great deal of experience (Becker *et al*., 2014; Börstler *et al*., 2014). To enable an unequivocal classification, several polymerase chain reaction (PCR)‐based assays have been developed using different molecular genetic markers (e.g. *ace‐2* or *ITS2*) (Smith & Fonseca, 2004; Vinogradova & Shaikevich, 2007). Rudolf *et al*. (2013) established a multiplex real‐time PCR for the simultaneous detection and differentiation of *Cx. pipiens* biotypes, *Cx. torrentium* and possible hybrids. In all cases, time‐consuming and cost‐intensive DNA extraction is necessary. The present study introduces a direct PCR technique that allows PCR amplification, as well as subsequent DNA sequencing without prior DNA extraction, for the identification of different mosquito species (Fig. 1). The relevant tissue can be used ‘directly’ as a template for amplification of the target gene or gene fragment. Usually, DNA extraction involves an overnight lysis of the tissue samples and an additional extraction step afterwards to obtain the template DNA for amplification. Using direct PCR, the time required can be reduced to a few minutes. The relevant tissue can be given to the prepared mastermix and amplified in a conventional PCR cycler. The establishment of cost‐efficient and time‐effective methods for the clear identification of distinct mosquito species is crucial to the success of surveillance programmes and must incorporate the fast identification of new and invasive species, as well as morphologically similar native species. This method was previously performed successfully to identify *Dirofilaria* species in mosquitoes (Silbermayr *et al*., 2013) and for malaria diagnosis (Fuehrer *et al*., 2011). In addition, direct PCR was carried out with different polymerases and primers by Wong *et al*. (2014) for the identification of non‐biting midges and other arthropods, including mosquitoes. In the present study, a variety of native and invasive mosquito species were amplified using direct PCR. Further, different amounts and storage conditions of tissue were analysed.

## Materials and methods

The present analyses were carried out using 62 specimens of 28 different mosquito species of 10 genera. The method was applied to mosquitoes which had been stored at −20, −80 °C or room temperature, or had been preserved in 70% ethanol or had been stored as dry material for between 1 month and 3 years. Different amounts of tissue were used to test the minimum amount of tissue required to produce positive PCR results in the gel electrophoresis. Barcoding with direct PCR was carried out with the Phusion^®^ Blood Direct PCR Kit (Thermo Fisher Scientific, Inc., Waltham, MA, U.S.A.). The reaction mixture (50 µL) consisted of 25 µL of direct PCR buffer, 1 µm of each primer and 1 U Phusion^®^ II DNA polymerase. Millipore water was added to a total volume of 50 µL, as were different tissue samples (not homogenized) as DNA template (Table 1). The Phusion^®^ high‐fidelity polymerase provided in the kit has a 50‐fold lower error rate than commercial standard *Taq* polymerases and is extremely resistant to inhibitors found in samples that are not extracted or purified before amplification [see #F‐547S (Thermo Fisher Scientific, Inc., Waltham, MA, U.S.A.) and #M0530S (New England Biolabs, Inc., Beverly, MA, U.S.A.)]. The amplification of a cytochrome c oxidase subunit 1 (COX1) gene fragment was performed in a thermocycler (Eppendorf Vertrieb Deutschland GmbH, Hamburg, Germany) using BC‐Kumar forward/reverse primers (Kumar *et al*., 2007). The parameters for PCR cycling were as follows: one cycle of 94 °C for 2 min; 40 cycles of 94 °C for 60 s, 59 °C for 60 s and 72 °C for 60 s, followed by a terminal extension of 72 °C for 5 min and a final ramping to 8 °C. The quality and yield of DNA were analysed by Midori Green (Nippon Genetic Europe GmbH, Düren, Germany) staining and agarose gel electrophoresis. Positive samples were purified using the peqGOLD Cycle‐Pure Kit (Peqlab Biotechnology GmbH, Erlangen, Germany) and Illustra™ ExoStar™ 1‐Step (GE Healthcare, Chalfont St Giles, U.K.). The subsequent Sanger sequencing was performed using Seqlab (Seqlab‐Sequence Laboratories Göttingen GmbH, Göttingen, Germany) and Microsynth (Microsynth AG, Balgach, Switzerland) using the BC‐Kumar forward and/or reverse primer. Each sequence was edited using BioEdit (Hall, 1999) and compared with sequences deposited in GenBank using the blast algorithm (Altschul *et al*., 1997).

**Table 1:** Culicidae used for species identification with direct polymerase chain reaction (PCR).

Serial no.	Genus	Species	Storage conditions	Storage time	Tissue used	PCR result	GenBank accession no.
1	*Aedes*	*Aedes cinereus* Meigen, 1818	70% EtOH rt	1 year	Leg (♀)	Positive	KM280590
2		*Aedes cinereus* Meigen, 1818	Dry	18 months	Leg (♀)	Negative	—
3		*Aedes cinereus* Meigen, 1818	Dry	18 months	Leg (♀)	Negative	—
4		*Aedes cinereus* Meigen, 1818	Dry	18 months	Leg (♀)	Negative	—
5	*Aedimorphus*	*Aedimorphus vexans* (Meigen, 1830)	Dry	18 months	Imago (♀)	Positive	KM280575
6		*Aedimorphus vexans* (Meigen, 1830)	Dry	18 months	Leg (♀)	Positive	KM280576
7		*Aedimorphus vexans* (Meigen, 1830)	−20 °C	2 years	Leg (♀)	Positive	KM243937
8	*Anopheles*	*Anopheles algeriensis* Theobald, 1903	Dry	18 months	Leg (♀)	Negative	—
9		*Anopheles algeriensis* Theobald, 1903	Dry	18 months	Leg (♀)	Negative	—
10		*Anopheles algeriensis* Theobald, 1903	Dry	18 months	Leg (♀)	Negative	—
11		*Anopheles algeriensis* Theobald, 1903	Dry	18 months	Leg (♀)	Negative	—
12		*Anopheles claviger s.s* (Meigen, 1804)	−20 °C	2 years	Leg (♀)	Positive	KM243938
13		*Anopheles gambiae s.s*. Giles, 1902	70% EtOH rt	6 months	Imago (♀)	Positive	Not sequenced
14		*Anopheles gambiae s.s*. Giles, 1902	70% EtOH rt	6 months	Leg (♀)	Positive	Not sequenced
15		*Anopheles gambiae s.s*. Giles, 1902	70% EtOH rt	6 months	Larva	Positive	Not sequenced
16		*Anopheles hyrcanus* (Pallas, 1771)	Dry	18 months	Imago (♀)	Positive	KM280592
17		*Anopheles hyrcanus* (Pallas, 1771)	Dry	18 months	Leg (♀)	Positive	KM280591
18		*Anopheles messeae* Falleroni, 1926	−20 °C	2 years	Leg (♀)	Positive	KM280597
19		*Anopheles plumbeus* Stephens, 1828	Dry	18 months	Leg (♀)	Positive	KM280577
20		*Anopheles plumbeus* Stephens, 1828	−20 °C	2 years	Leg (♀)	Positive	KM243939
21	*Coquillettidia*	*Coquillettidia richiardii* (Ficalbi, 1889)	−20 °C	2 years	Leg (♀)	Positive	KM243940
22		*Coquillettidia richiardii* (Ficalbi, 1889)	−20 °C	2 years	Leg (♀)	Positive	KM243941
23	*Culex*	*Culex modestus* Ficalbi, 1889	Dry	18 months	Leg (♀)	Positive	KM280578
24		*Culex pipiens molestus* Forskal, 1775	−20 °C	2 years	Leg (♀)	Positive	KM243942
25		*Culex pipiens molestus* Forskal, 1775	−20 °C	2 years	Leg (♀)	Positive	KM243943
26		*Culex pipiens molestus* Forskal, 1775	−20 °C	2 years	Leg (♀)	Positive	KM243944
27		*Culex pipiens molestus* Forskal, 1775	−20 °C	2 years	Leg (♀)	Positive	KM243945
28		*Culex pipiens molestus* Forskal, 1775	−20 °C	2 years	Leg (♀)	Positive	KM243946
29		*Culex pipiens molestus* Forskal, 1775	−20 °C	2 years	Leg (♀)	Positive	KM243947
30		*Culex pipiens pipiens* Linnaeus, 1758	−80 °C	6 months	Imago (♀)	Positive	KM280595
31		*Culex pipiens pipiens* Linnaeus, 1758	−80 °C	6 months	Imago (♀)	Positive	KM280596
32		*Culex pipiens pipiens* Linnaeus, 1758	70% EtOH rt	18 months	Leg (♀)	Positive	KM280594
33		*Culex territans* Walker, 1856	Dry	18 months	Head (♀)	Positive	KM280581
34		*Culex torrentium* Martini, 1925	−20 °C	2 years	Leg (♀)	Positive	KM243948
35		*Culex quinquefasciatus* Say, 1832	70% EtOH rt	6 months	Imago (♀)	Positive	KM280579
36		*Culex quinquefasciatus* Say, 1832	70% EtOH rt	6 months	Leg (♀)	Positive	KM280580
37	*Culiseta*	*Culiseta annulata* (Schrank, 1776)	−80 °C	1 month	Leg (♀)	Positive	KM280582
38		*Culiseta annulata* (Schrank, 1776)	70% EtOH rt	18 months	Leg (♀)	Positive	KM280593
39	*Hulecoenteomyia*	*Hulecoeteomyia j. japonica* (Theobald, 1901)	−20 °C	1 year	Leg (♀)	Positive	KM243953
40		*Hulecoeteomyia j. japonica* (Theobald, 1901)	−20 °C	1 year	Leg (♀)	Positive	KM243954
41	*Ochlerotatus*	*Ochlerotatus cantans* (Meigen, 1818)	70% EtOH rt	18 months	Larva	Positive	not sequenced
42		*Ochlerotatus caspius* (Pallas, 1771)	Dry	18 months	Head (♀)	Positive	KM280583
43		*Ochlerotatus caspius* (Pallas, 1771)	−20 °C	1 year	Leg (♀)	Positive	KM243949
44		*Ochlerotatus caspius* (Pallas, 1771)	−20 °C	1 year	Leg (♀)	Positive	KM243950
45		*Ochlerotatus cataphylla* (Dyar, 1916)	−20 °C	1 year	Leg (♀)	Positive	KM243951
46		*Ochlerotatus excrucians* (Walker, 1856)	−20 °C	2 years	Leg (♀)	Positive	KM243952
47		*Ochlerotatus flavescens* (Müller, 1764)	Dry	18 months	Leg (♀)	Positive	Not sequenced
48		*Ochlerotatus geniculatus* (Olivier, 1791)	Dry	18 months	Leg (♀)	Positive	KM280584
49		*Ochlerotatus punctor* (Kirby, 1837)	−20 °C	1 year	Leg (♀)	Positive	KM243955
50		*Ochlerotatus rusticus* (Rossi, 1790)	Dry	3 years	Leg (♀)	Positive	KM243956
51		*Ochlerotatus rusticus* (Rossi, 1790)	Dry	3 months	Leg (♀)	Positive	KM243957
52		*Ochlerotatus sticticus* (Meigen, 1838)	Dry	18 months	Leg (♀)	Positive	KM280585
53		*Ochlerotatus sticticus* (Meigen, 1838)	Dry	18 months	Leg (♀)	Positive	KM280586
54		*Ochlerotatus sticticus* (Meigen, 1838)	Dry	18 months	Leg (♀)	Positive	KM280587
55		*Ochlerotatus sticticus* (Meigen, 1838)	−20 °C	2 years	Leg (♀)	Positive	KM243958
56		*Ochlerotatus sticticus* (Meigen, 1838)	70% EtOH rt	1 year	Leg (♀)	Positive	KM243959
57		*Ochlerotatus sticticus* (Meigen, 1838)	70% EtOH rt	1 year	Leg (♀)	Positive	KM243960
58	*Stegomyia*	*Stegomyia aegypti* (Linnaeus, 1762)	70% EtOH rt	6 months	Imago (♀)	Positive	KM280573
59		*Stegomyia aegypti* (Linnaeus, 1762)	70% EtOH rt	6 months	Leg (♀)	Positive	KM280574
60		*Stegomyia aegypti* (Linnaeus, 1762)	−20 °C	1 year	Leg (♀)	Positive	KM243936
61	*Uranotaenia*	*Uranotaenia unguiculata* Edwards, 1913	Dry	18 months	Leg (♀)	Positive	KM280588
62		*Uranotaenia unguiculata* Edwards, 1913	Dry	18 months	Leg (♀)	Positive	KM280589

rt, room temperature.

## Results

The direct PCR method was successfully performed in 55 individuals belonging to 28 different mosquito species, both indigenous and non‐indigenous to Austria and Germany. Seven samples of two species showed no positive results after amplification and gel electrophoresis (Table 1). In almost all cases, the use of one leg (*n* = 44) as DNA template was sufficient to generate PCR products of an expected size of ≈ 700 bp. Furthermore, tissue samples such as heads (*n* = 2), larvae (*n* = 2) or imagoes (*n* = 7) were successfully used as templates. Tissue stored under different conditions (−20, −80 °C, 70% ethanol or dry) also produced positive results. Storage times for the specimens analysed varied between 1 month and 3 years and did not seem to have significant influence. However, dried legs of *Aedes cinereus* Theobald, 1901 and *Anopheles algeriensis* Theobald, 1903 stored for 18 months at room temperature did not yield positive PCR results. Thus, the samples could not be sequenced. The genera *Ochlerotatus* (nine species) and *Culex* (six species) showed the best amplification results obtained with this method. To verify the correct amplification of the COX1 gene fragments by direct PCR and to subsequently differentiate the analysed species, most of the positive samples were purified and sequenced. The data obtained have been deposited at GenBank under the accession numbers KM243936–KM243960 (Germany) and KM280573–KM280597 (Austria) (Table 1). Positive individuals of *Anopheles gambiae s.s*. Giles, 1902, *Ochlerotatus cantans* (Meigen, 1818) and *Ochlerotatus flavescens* (Müller, 1764) were not sequenced (Table 1).

## Discussion

As a variety of non‐native mosquito species can be considered as competent vectors for both endemic and newly introduced or emerging pathogens, their introduction is of increasing public and political concern in numerous countries, including Austria and Germany (Avšič‐Županc, 2013; Becker *et al*., 2014). Both recently introduced and indigenous mosquito species, such as members of the *Cx. pipiens* or *An. maculipennis* complexes, are able to transmit a broad range of pathogens, and are difficult to distinguish morphologically (Farajollahi *et al*., 2011; Laboudi *et al*., 2014). Nevertheless, it is essential that mosquitoes are clearly identified in order to generate information on their distribution and the potential risk for the transmission of disease. The direct PCR method proposed in this study offers a time‐ and cost‐effective amplification technique for mosquito specimen analysis. Based on the PCR results, subsequent sequencing and thus the identification of different mosquito species is possible. The greatest advantage of this method in comparison with standard PCR techniques for species identification is its facilitation of the amplification of DNA from tissue without prior DNA extraction; the non‐homogenized tissue is simply mixed with PCR reagents and transferred to a thermal cycler for amplification. Depending on the DNA extraction and amplification techniques used as standard in various laboratories, direct PCR can reduce the time to amplify target genes for species identification. In the present study, sequencing of the samples was carried out after 5 h instead of 1 day because the process eliminated the need for overnight lysis. Additionally, the method is not directly affected by the specimens' morphological state of preservation. Important morphological characters are frequently lost during sampling, transportation or storage. Without these characters it is extremely difficult to identify the different mosquito species. One of the parameters important to the production of positive PCR results is the amount of tissue used, which can lead to PCR failures during the initial heating step (Wong *et al*., 2014). Wong *et al*. (2014) used two or three legs of each individual to obtain a positive amplification result. In the present study, a head or one leg was sufficient to produce positive results and allowed for the preservation of important structures for morphological reference collections. The direct use of tissue is a limitation of this method as further analyses with the PCR product are not possible (e.g. amplification of different markers) and no extracted DNA is available for storage. However, this method is useful for rapid species identification, especially of new vector species, based on the fast amplification of the target gene. If further analyses are needed, DNA extraction of the remaining tissue is still possible. Moreover, RNA extraction from the remaining tissue is possible, which is important as many species are carriers of disease agents such as flaviviruses (e.g. WNV), alphaviruses (e.g. Sindbis virus) and orthobunyaviruses (e.g. Batai virus). In order to test how much tissue can be introduced into direct PCR, whole larvae or adults were successfully used as templates; however, Wong *et al*. (2014) warn that abdominal tissue should be avoided as too many inhibitors from intestines may be present. In addition to different amounts of tissue, outcomes in various conditions and periods of storage were examined. DNA amplification from tissue stored at −20 °C (*n* = 22), −80 °C (*n* = 3) and in 70% ethanol (*n* = 13) is relatively easy. However, amplification from dried specimens (*n* = 24) may be difficult. In total, 22 of 24 dry samples used had been stored for 18 months. Seven of these samples belonging to the species *Ae. cinereus* (*n* = 3) and *An. algeriensis* (*n* = 4) gave no positive PCR result and therefore could not be sequenced. In order to avoid the failure of the direct PCR, the legs of the two species were analysed using conventional DNA extraction and amplification methods, which also produced negative results. Additionally, direct PCR as well as normal PCR with previously extracted DNA from whole adults of both species revealed positive bands after gel electrophoresis. The negative direct PCR results using legs as templates could be attributed to the conservation of the specimens. For the prevention of collection pests, dry preparations have often been treated with the insecticide dichlorvos, which affects the DNA of insects negatively (Espeland *et al*., 2010). Other parameters that may influence the amplification are the primer pairs and the polymerase used. First, the standard barcoding primers by Folmer *et al*. (1994), which were also successfully applied by Wong *et al*. (2014), were used. However, the amplification results showed great variances and a blast search revealed that the primers, because of their low specificity, are unsuited to DNA barcoding of mosquitoes using direct PCR. To overcome this issue, Kumar *et al*. (2007) designed a primer set that was then applied in the present study. The polymerase used in this study has extremely high resistance to inhibitors and shows a much lower error rate than standard *Taq* polymerases. The cost‐effectiveness of this technique for a normal sample work‐off (single samples, not pooled) per reaction can be shown by the price per sample analysed. Obviously, saving money depends on the techniques, kits and research issues that pertain to different laboratories. However, in the present study, using the Phusion^®^ Blood Direct PCR Kit, the analysis of one sample was estimated to cost €1.83. By contrast, a conventional DNA extraction and PCR costs about €3.36 per sample [extraction: peqGOLD Tissue DNA Mini Kit (S‐Line), €2.05/reaction; PCR: peqGOLD Hot Start Mix Y, €1.31/reaction; both Peqlab Biotechnology GmbH].

## Conclusions

The use of direct PCR represents a rapid method of amplifying genes or gene fragments that can be used for the clear identification of mosquito species after a subsequent sequencing reaction. The method is ideal for the analysis of native and non‐native mosquito species, especially in the context of uncertain morphological determination. Moreover, compared with the standard DNA extraction and amplification kits used, direct PCR proved to be much cheaper in the present study. Overall, the ability to save time is of benefit in modern laboratory work, especially in the context of the identification of invasive species because it can facilitate rapid monitoring and control in the event of positive findings. Although this study is not the first to use direct PCR for the identification of mosquitoes, its findings include results obtained from analyses of different amounts of tissue, as well as in contexts of different conditions and periods of storage, for 28 different mosquito species.
